# Physician documented fatigue among adults and children with inflammatory bowel disease: a nationwide routinely collected health data study from the epi-IIRN

**DOI:** 10.1093/crocol/otag116

**Published:** 2026-10-12

**Authors:** Gili Focht, Rachel Buchuk, Menachem Sokolik, Shlomi Cohen, Henit Yanai, Amir Ben-Tov, Galia Zacay, Eran Matz, Shir Zekaria, Iris Dotan, Ora Paltiel, Dan Turner

**Affiliations:** Juliet Keidan Institute of Pediatric Gastroenterology Hepatology and Nutrition, The Eisenberg R&D Authority, Shaare Zedek Medical Center, The Hebrew University of Jerusalem, Jerusalem, Israel; Braun School of Public Health and Community Medicine, Faculty of Medicine, Hebrew University of Jerusalem, Jerusalem, Israel; Juliet Keidan Institute of Pediatric Gastroenterology Hepatology and Nutrition, The Eisenberg R&D Authority, Shaare Zedek Medical Center, The Hebrew University of Jerusalem, Jerusalem, Israel; Juliet Keidan Institute of Pediatric Gastroenterology Hepatology and Nutrition, The Eisenberg R&D Authority, Shaare Zedek Medical Center, The Hebrew University of Jerusalem, Jerusalem, Israel; Pediatric Gastroenterology Institute, “Dana-Dwek” Children’s Hospital, Tel Aviv Sourasky Medical Center, Tel Aviv, Israel; Gray Faculty of Medical & Health Sciences, Tel Aviv University, Tel Aviv, Israel; Gray Faculty of Medical & Health Sciences, Tel Aviv University, Tel Aviv, Israel; IBD Center, Division of Gastroenterology, Rabin Medical Center, Petah Tikva, Israel; Gray Faculty of Medical & Health Sciences, Tel Aviv University, Tel Aviv, Israel; Maccabi Research and Innovation Center Maccabi Healthcare Services, Tel Aviv, Israel; Gray Faculty of Medical & Health Sciences, Tel Aviv University, Tel Aviv, Israel; Meuhedet Health Services, Tel Aviv, Israel; Leumit Health Services, Tel Aviv, Israel; Clalit Health Services, Clalit Research Institute, Tel Aviv, Israel; IBD Center, Division of Gastroenterology, Rabin Medical Center, Petah Tikva, Israel; Braun School of Public Health and Community Medicine, Faculty of Medicine, Hebrew University of Jerusalem, Jerusalem, Israel; Department of Hematology, Hadassah Medical Organization, Jerusalem, Israel; Juliet Keidan Institute of Pediatric Gastroenterology Hepatology and Nutrition, The Eisenberg R&D Authority, Shaare Zedek Medical Center, The Hebrew University of Jerusalem, Jerusalem, Israel

**Keywords:** fatigue, Crohn’s disease, ulcerative colitis, nationwide, epi-IIRN

## Abstract

**Background:**

We aimed to assess the rates of physician-documented fatigue according to diagnostic codes among patients with inflammatory bowel diseases (IBDs) in a nationwide cohort and to identify clinical factors associated with its documentation.

**Methods:**

We used the nationwide epi- Israeli IBD Research Nucleus cohort derived from 4 HMOs covering approximately 98% of the Israeli population, matched to non-IBD controls. Physician-documented fatigue was defined using routinely collected ICD9 and textual codes and explored in association with demographic, laboratory-derived disease severity clusters, comorbidities, medications, healthcare utilization, and sickness absence. Predictors for fatigue documentation were explored using multivariable Cox models incorporating time-varying covariates.

**Results:**

Fatigue was documented in 16.4% of the 31 945 patients with IBD and 11.0% of the 73 106 matched controls (odds ratio [OR] = 1.60, 95% confidence interval [CI] 1.54-1.66). Fatigue was more prevalent in IBD than matched controls across age groups and comorbidity strata. Among patients with IBD, there was no difference in fatigue rate between those with Crohn’s disease and ulcerative colitis. Time-to-fatigue was shorter among females (*P *< .001). Across adult males and females and pediatric boys and girls, anemia, anxiety, and ≥3 healthcare contacts were independently associated with higher rates of fatigue documentation, with the strongest associations observed for frequent healthcare contact (HRs ranging from 4.21 [95% CI 1.49-11.70] to 10.22 [95% CI 7.47-13.90]). Longer disease duration was consistently associated with a lower hazard, while additional subgroup-specific associations were observed for sleep disorders, steroid dependency, and IBD-related medications.

**Conclusions:**

Fatigue is more frequently documented among patients with IBD than matched non-IBD controls, especially in females. Several risk factors were associated with fatigue which should be considered in clinical practice. The lower frequency of documented fatigue compared with patient-reported estimates suggests that routine clinical coding captures only part of the overall fatigue burden.

Key Messages ***What is already known?*** 

- Fatigue is a highly prevalent, debilitating symptom in patients with inflammatory bowel disease, driven by different facets, including active inflammation, anemia, possible nutritional deficiencies and psychological comorbidities.

***What is new here?*** 

- This study evaluates physician-documented fatigue within an electronic medical record setting across an unbiased, unselected nationwide cohort.

***How can this study help patient care?*** 

- By demonstrating that medical records capture only the “tip of the iceberg” of fatigue burden, these findings encourage clinicians to proactively screen inflammatory bowel disease patients for fatigue during routine visits.

## Introduction

Inflammatory bowel diseases (IBDs) are associated with multiple physiological and psychosocial manifestations, including fatigue, which may be more common than recognized in the literature.^1–4^ Recent systematic reviews have shown that the rate of fatigue ranges from 24% to 87% in adults^5^ and from 22% to 78% in children.^6–8^ Patients frequently report fatigue as one of the more burdensome presentations of IBD, even more than gastrointestinal symptoms.^9^ In a recent study, children and adolescents with IBD rated fatigue (causes and steps for reduction) as the number one research priority of a list of 10 topics, higher than topics including nutrition and gut microbiome and personalized medication.^10^

Fatigue is defined as “a persistent, overwhelming sense of tiredness, weakness or exhaustion resulting in decreased capacity for physical and/or mental work”.^11^ There are a number of suggested causes of fatigue in IBD, notably anemia, active disease, poor sleep quality, iron and other micronutrient deficiencies as well as associated disorders (eg, celiac disease, and medication-induced Lupus erythematosus). Fatigue has also been reported in 41% of those in complete remission without any apparent organic cause,^7^^,^^8^^,^^12^ and may be present many years after diagnosis.^13^ Psychological aspects and brain-gut axis interactions may contribute to fatigue in IBD, although these mechanisms remain poorly understood.^2^^,^^14–16^

Current estimates of the rates of fatigue are almost exclusively based on questionnaires completed by patients. In the absence of prior studies examining fatigue as documented in electronic medical records, we aimed to assess the rates of physician-documented fatigue among children and adults with IBD in a nationwide, unselected administrative cohort, based on electronic medical records, and to identify possible clinical features associated with this phenotype. Reporting physician-documented fatigue provides insight into how often fatigue is recognized and formally recorded during routine clinical care, possibly assuming that more clinically relevant or severe cases will be documented during physician visits.

## Materials and methods

This study utilized the validated Israeli IBD Research Nucleus (epi-IIRN) cohort,^17^ a nationwide database comprising patients with IBD from the 4 national Health Maintenance Organizations (HMOs), which collectively cover approximately 98% of the Israeli population (*n* = 58 640 as of June 30, 2021). The HMOs are fully computerized and maintain electronic medical records, including laboratory tests, imaging, diagnosis, medications, surgeries and other ambulatory health services linked online to a central server. Medication data are accurate since they are covered by the national health system and are dispensed by the HMOs. ICD9 and textual codes are recorded by the treating physicians, nurses and allied health professionals, during routine visits. The development and validation of the IBD case ascertainment algorithm have been previously described.^17^ Briefly, the algorithm, utilizing a combination of IBD-related ICD9 codes and medications, demonstrated high predictive value (specificity 99%, sensitivity 89%, negative predictive value [NPV] 99% and positive predictive value [PPV] 92%). IBD type is determined by the majority of Crohn’s disease (CD)/ulcerative colitis (UC) specific codes out of the 3 most recent healthcare contacts, or the most recent when <3 are recorded (sensitivity 92%, specificity 97%, PPV 97% and NPV 92%).^17^ Since there is no IBD-unclassified diagnostic code in the HMO database, patients are classified as CD or UC using the validated epi-IIRN disease-type algorithm.^17^

We included patients of all ages with newly diagnosed IBD since 2005 (previously validated as the cutoff year for determining incidence in Israeli HMOs^17^) followed up to July 2020 (to allow time to acquire fatigue related codes). Children born after 2000 were also included even if their IBD was diagnosed before 2005 (since the database covers their entire life span and thus there is no need for a lookback period). Each patient with IBD was randomly matched with 2-3 non-IBD controls based on year of birth, sex, district and HMO. No additional matching criteria were applied. For the overall cohort, individuals with fatigue documented >2 years before the index date were excluded, as fatigue may represent an early manifestation of IBD given frequent diagnostic delays^18^^,^^19^ and considering that the date of diagnosis in the epi-IIRN cohort is accurate within 1 year of the recorded date. For time-to-event analyses, individuals with any fatigue code before the index date were additionally excluded.

Physician-documented fatigue (fatigue) was defined as having at least one of the following ICD9 codes: “Malaise and fatigue” (780.7), “Chronic Fatigue” (780.71) or textual codes of “fatigue” within diagnosis fields (Table S1 for all included codes). “Pregnancy related fatigue” and “heat fatigue” codes were excluded. The fatigue coding algorithm was not formally validated against detailed chart review or a validated patient-reported fatigue measure. The rate of recorded fatigue codes among the patients with IBD was compared with that among matched non-IBD controls. In addition, to assess the potential misclassification arising from isolated diagnostic codes, we performed a sensitivity analysis using a more stringent definition requiring at least 2 fatigue-related codes at 2 separate visits. To examine whether IBD is associated with an independent risk of fatigue exceeding the burden of general chronic illness, rates of recorded fatigue codes among the IBD cohort were compared to the matched controls, stratified separately by the presence or absence of comorbidities. Comorbidities were derived from routinely recorded diagnostic codes and updated at each disease quarter; no additional clinical assessment was performed for the study. In adults, comorbidity burden was classified using the Charlson’s Comorbidity Index (CCI,^20^ categorized as 0 indicating no comorbidities, 1-2 as mild, 3-4 as moderate and ≥5 as severe^21^). In children, comorbidities were classified using the Pediatric Chronic Complex Conditions (CCCs).^22^ At each disease quarter, newly recorded comorbid diagnoses were incorporated into the patient’s comorbidity profile, and the CCI/CCC classification was updated accordingly. In addition, we assessed the rates of specific comorbidities associated with fatigue, such as anxiety, depression and sleep disorders by exploring the use of these ICD9 codes within the electronic medical records. All codes were assessed based on the routine clinical records. Absence of a code was interpreted as absence of documented disease rather than confirmed absence of the condition or missingness. To assess whether laboratory-derived disease severity at diagnosis was associated with subsequent fatigue, we used severity clusters based on available blood tests around the time of IBD diagnosis (hemoglobin, C-reactive protein, erythrocyte sedimentation rate, albumin, platelets, white blood cell count) as developed and validated in the epi-IIRN and external cohorts.^23^^,^^24^ This approach effectively handles missing data while performing hierarchical clustering to group the population based on phenotypic similarities. These clusters were not updated annually because laboratory data became progressively less complete with increasing time from diagnosis. To assess the possible influence of IBD medications on fatigue, we defined the use of medication at each disease quarter, ordered hierarchically (Table S2). This included the use of IBD-related medication and steroid-dependency, which was defined as cumulative use of systemic corticosteroids for >90 days in a calendar year. Anemia and iron deficiency were defined based on diagnostic codes (both ICD9 and textual, Table S3); and recorded fatigue was explored in relation to documentation of anemia. We also used both the number of healthcare contacts during each disease quarter starting from the year of IBD diagnosis, and the number of approved sickness absence days, issued by the physician, as measures of healthcare utilization and overall health burden.^25^^,^^26^ To assess potential ascertainment bias, we compared the distribution of physician specialties assigning fatigue codes between IBD patients and matched controls.

### Statistical analyses

Descriptive data are presented as means ± standard deviation or medians (interquartile range [IQR]), as appropriate. To explore univariable differences between groups, we used the standardized mean difference (SMD) for continuous variables and odds ratio (OR), with 95% confidence interval (CI) for categorical variables and rates. SMD is preferred in samples with a large size as it reflects the effect size and not merely statistical significance.^27^ SMD was considered clinically significant when >0.1.^28^ Incidence was calculated using person-time rates. Time to first fatigue code from IBD diagnosis was compared using the log-rank test, using the IBD diagnosis date at the starting timepoint for the matched controls (those with fatigue codes prior to diagnosis of IBD were excluded from this analysis). To assess the association between comorbidities (CCI) and fatigue while accounting for the longitudinal nature of the data, we utilized a generalized estimating equation (GEE) model^29^ with a logit link function and an exchangeable working correlation structure. GEE accounts for repeated measures within the same patient over time (quarters). This method uses all available data points to estimate the population-averaged OR, while robust standard errors are calculated to adjust for within-subject correlations. Cox proportional hazards models were constructed to identify independent predictors of fatigue. Comorbidity status (CCI/CCC per age group), fatigue related comorbidities (such as anxiety, depression etc.), number of healthcare contacts, and medication exposure were updated at each disease quarter and modeled as time-varying covariates. Pediatric analyses were additionally stratified by sex using the same approach. The proportional hazards assumption was verified using the cox.zph function from the survival package. Analysis was performed in R version 4.4.1.

## Results

### IBD versus non-IBD control

A total of 31 945 patients with IBD were matched with 73 106 non-IBD controls with similar follow-up time (7.6 [IQR 3.8-11.8] years vs. 7.5 [IQR 3.8-11.7] years; SMD < 0.01, *P *= .34, Table 1). Fatigue was documented (ever/never) in 5247 (16.4%) of patients with IBD compared to 8011 (11%) of the non-IBD controls (OR 1.60 [95% CI 1.54-1.66]). These results were similar across all stratified age groups (children through elderly) ranging from 8.9%-23.4% among the patients with IBD compared to 4.8%-17.3% in the matched non-IBD controls (ORs 1.46-1.90, [95% CI 1.31-2.16], Table 1).

**Table 1:** Rates of physician-documented fatigue code in patients with inflammatory bowel disease (IBD) compared to non-IBD matched controls (counts [%], median [interquartile range] or mean ± SD are presented as appropriate).

	IBD cases	Non-IBD controls	**SMD** ^a^	Odds ratio (95**%** confidence interval)
***N***	31 945	73 106	—	—
**Sex (male)**	16 965 (53.1%)	38 319 (52.4%)	0.014	—
**Follow up, years**	7.58 (3.80, 11.81)	7.51 (3.78, 11.73)	0.007	—
**Socioeconomic status**	6.01 ± 2.00	5.58 ± 2.12	0.209	—
**Number of individuals with fatigue related code**	5247 (16.4%)	8011 (11.0%)	—	1.60 (1.54-1.66)
** Pediatric (0-17 years)**	469 (8.9%)	518 (4.8%)	—	1.90 (1.67-2.16)
** Young adults (18-40 years)**	2633 (16.5%)	4225 (11.5%)	—	1.51 (1.43-1.59)
** Adults (41-64 years)**	1478 (18.6%)	2076 (10.9%)	—	1.83 (1.7-1.97)
** Elderly (≥65 years)**	667 (23.4%)	1192 (17.3%)	—	1.46 (1.31-1.62)
**Comorbidities by CCI** ^b^				
** None (Charlson’s Comorbidity Index [CCI] = 0)**	9871 (32.4%)	32 566 (45.8%)	—	1.68 (1.60-1.76)
** Mild (CCI = 1-2(**	6582 (21.6%)	17 981 (25.3%)	—	1.72 (1.62-1.83)
** Moderate (CCI—3-4)**	7302 (24.0%)	8643 (12.2%)	—	1.68 (1.56-1.81)
** Severe (CCI ≥5)**	6732 (22.1%)	11 938 (16.8%)	—	1.17 (1.09-1.25)
**Comorbidities categories at diagnosis (top 5 contributing categories in IBD)**				
** Chronic pulmonary disease**	5486 (17.2%)	10 457 (14.3%)	0.079	—
** Rheumatologic disease**	2747 (8.6%)	6105 (8.4%)	0.009	—
** Peptic ulcer disease**	2249 (7.0%)	3115 (4.3%)	0.121	—
** Controlled diabetes**	1814 (5.7%)	4403 (6.0%)	0.015	—
** Malignancy (any)**	1507 (4.7%)	2870 (3.9%)	0.039	—

a SMD (standardized mean difference) reflects the effect size and not only statistical significance which is more appropriate in large datasets. SMD is considered significant when >0.1.^27^^,^^28^

b CCI—Charlson’s Comorbidity Index, stratified into 4 categories—none, mild, moderate and severe^20^^,^^21^ Percentages reflect the distribution of the cohort across CCI strata.

Time to fatigue documentation was shorter in both female and male patients with IBD than in their matched non-IBD controls (Figure 1A and B); females with IBD showed the shortest time to fatigue documentation. In the pediatric population, time to fatigue was shorter in the IBD population in boys. When restricting the analysis to individuals with at least 2 fatigue-related codes, time to the 2nd fatigue code remained significantly shorter among patients with IBD compared with their matched controls in both pediatric and adult populations (*P *< .001). To explore ascertainment bias in the source of fatigue documentation, we compared the rates of fatigue codes given by primary physicians and specialists in both patients with IBD and non-IBD controls, which were similar (Figure S1). To address the potential bias that patients with IBD may have a higher burden of comorbidities independent of any causal relationship between IBD and fatigue, we stratified both the IBD and the non-IBD cohorts by CCI score. While fatigue rates increased alongside comorbidity severity in both groups (IBD and non-IBD), fatigue remained significantly more prevalent among patients with IBD across all comorbidity strata (ORs ranging from 1.17 [95% CI 1.09-1.25] for severe comorbidity to 1.72 [95% CI 1.62-1.83] for mild comorbidities).

**Figure 1:**
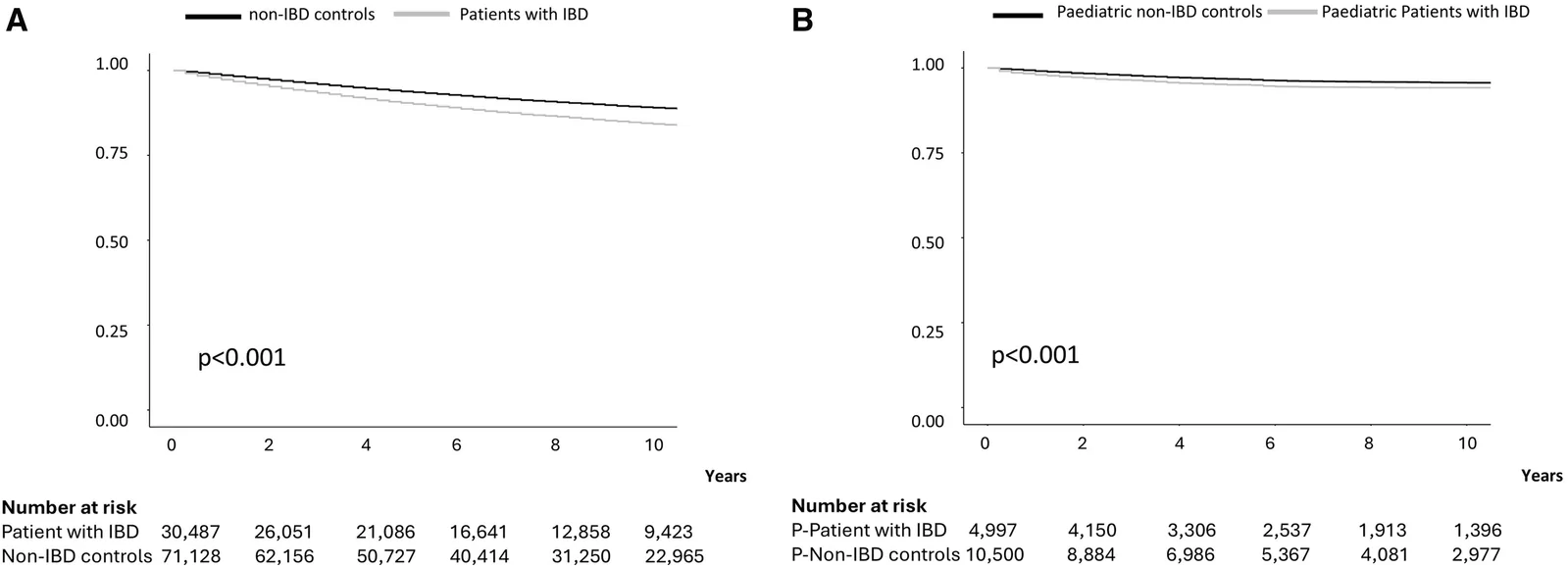
Time to first physician documented fatigue code in patients with inflammatory bowel disease (IBD) and non-IBD controls (A: All; B: pediatric).

### Fatigue within the IBD population

There were no differences in documented fatigue rates (16.1% vs. 16.7%, *P *= .19) or time to documented fatigue (*P *= .57, Figure S2) between CD and UC. Time to fatigue was shorter in females than in males with IBD (*P *< .001, Figure S3). This was not observed within children with IBD, who had similar time to fatigue among girls and boys (*P *= .15).

#### Predictors of fatigue documentation

In univariate analyses, older age, anemia, iron deficiency, anxiety, depression, sleep disorders, and increased number of sickness absence days and healthcare visits were associated with fatigue (Table 2). Of note, 11.8% of fatigue diagnoses occurred within ±3 months of anemia diagnosis.

**Table 2:** Physician-documented fatigue and other related comorbidities among the inflammatory bowel disease (IBD) population (numbers [%], median [interquartile range] or means ± standard deviations are presented as appropriate).

Variable	**Patients with IBD without physician documented fatigue (*n* = 26** **698)**	Patients with IBD with physician documented fatigue (*n* = 5247)	**SMD** ^a^	OR (95**%** CI)
**IBD subtype, Crohn’s disease**	15 007 (56.2%)	3002 (57.2%)	0.020	—
**Sex (male)**	14 642 (54.8%)	2323 (44.3%)	0.213	—
**Age at diagnosis**	34.73 ± 18.40	38.55 ± 18.80	0.205	—
**Follow up years**	7.13 (3.47, 11.46)	9.79 (6.02, 13.20)	0.422	—
**Sickness absence days** ^b^	78.32 ± 95.60	114.40 ± 128.20	0.319	—
**Social economic status**	6.00 ± 2.01	6.11 ± 1.97	0.058	—
**Anemia (ever/never)**	7132 (26.7%)	1983 (37.8%)	—	1.67 (1.57-1.77)
**Anxiety (ever/never)**	5018 (18.8%)	1721 (32.8%)	—	2.11 (1.98-2.25)
**Depression (ever/never)**	1830 (6.9%)	461 (8.8%)	—	1.31 (1.18-1.46)
**Iron deficiency (ever/never)**	8745 (32.8%)	1837 (35.0%)	—	1.11 (1.04-1.18)
**Sleep disorder (ever/never)**	1566 (5.9%)	686 (13.1%)	—	2.41 (2.19-2.65)
**Disease severity at diagnosis** ^c^			0.046	—
** Mild**	8784 (32.9%)	1658 (31.6%)	—	1.06 (1.00-1.13)
** Moderate**	15 368 (57.6%)	3134 (59.7%)	—	1.11 (1.00-1.23)
** Severe**	2546 (9.5%)	455 (8.7%)	—	0.91 (0.86-0.97)
**Healthcare contacts** ^d^	2.54 (0.00, 13.00)	11.90 (1.01, 21.58)	0.494	—

a SMD (standardized mean difference) reflects the effect size and not only statistical significance which is more appropriate in large datasets. SMD is considered significant when >0.1.^27^^,^^28^

b Starting 2 years prior to IBD diagnosis until the end of follow-up.

c By clustering analysis of blood tests available at diagnosis.^24^

d Assessed by the annual mean of all clinic visits.

The effect of sex violated the proportional hazards assumption of the Cox proportional model, and, therefore, the model was stratified by sex, while adjusting for disease type, disease duration, age, socioeconomic status (SES), comorbidity (CCI: mild/moderate/severe vs. none), number of healthcare contacts (ie, none, 1-2, ≥3), other diagnoses potentially associated with fatigue (depression, anxiety, sleep disorders, anemia, and iron deficiency) and IBD-related medications. Among males, anemia (HR 1.48, 95% CI 1.25-1.73), anxiety (HR 1.46, 95% CI 1.27-1.67), sleep disorders (HR 1.39, 95% CI 1.15-1.66), 1-2 healthcare contacts (HR 3.18, 95% CI 2.27-4.44), ≥3 healthcare contacts (HR 10.22, 95% CI 7.47-13.90), and steroid-dependency (HR 1.90, 95% CI 1.57-2.30) were independently associated with fatigue. Lower HRs were observed in longer disease duration (HR 0.98, 95% CI 0.96-0.99), and all other IBD related medications (HRs 0.60-0.83, 95% CIs 0.43-0.93) (Figure 2). Among the female patients, though the model violated the proportional hazards assumption (GLOBAL *P *= .04); anemia (HR 1.47, 95% CI 1.28-1.68), anxiety (HR 1.54, 95% CI 1.37-1.72), 1-2 healthcare contacts (HR 1.47, 95% CI 1.09-1.96), ≥3 healthcare contacts (HR 4.85, 95% CI 3.76-6.25), and steroid dependency (HR 1.44, 95% CI 1.17-1.76) were independently associated with fatigue. Lower HRs were observed in longer disease duration (HR 0.97, 95% CI 0.96-0.98), and all other IBD related medications (HRs 0.69-0.83, 95% CI 0.51-0.99) (Figure 3). Among the pediatric population, in boys, anxiety (HR 3.06, 95% CI 1.62-5.77), anemia (HR 1.86, 95% CI 1.06-3.25), and ≥3 healthcare contacts (HR 4.21, 95% CI 1.49-11.70), were independently associated with fatigue. Lower HRs were observed in longer disease duration (HR 0.95, 95% CI 0.90-0.99), and the use of 5-*aminosalicylic acid* (HR 0.52, 95% CI 0.29-0.92). Among girls, anxiety (HR 4.14, 95% CI 2.38-7.17), anemia (HR 3.21, 95% CI 1.92-5.33), and ≥3 healthcare contacts (HR 7.21, 95% CI 1.74-29.70), were independently associated with fatigue. As in boys, Lower HRs were observed in longer disease duration (HR 0.94, 95% CI 0.89-0.99), and the use of 5-*aminosalicylic acid* (HR 0.54, 95% CI 0.31-0.95) (Table S5).

**Figure 2:**
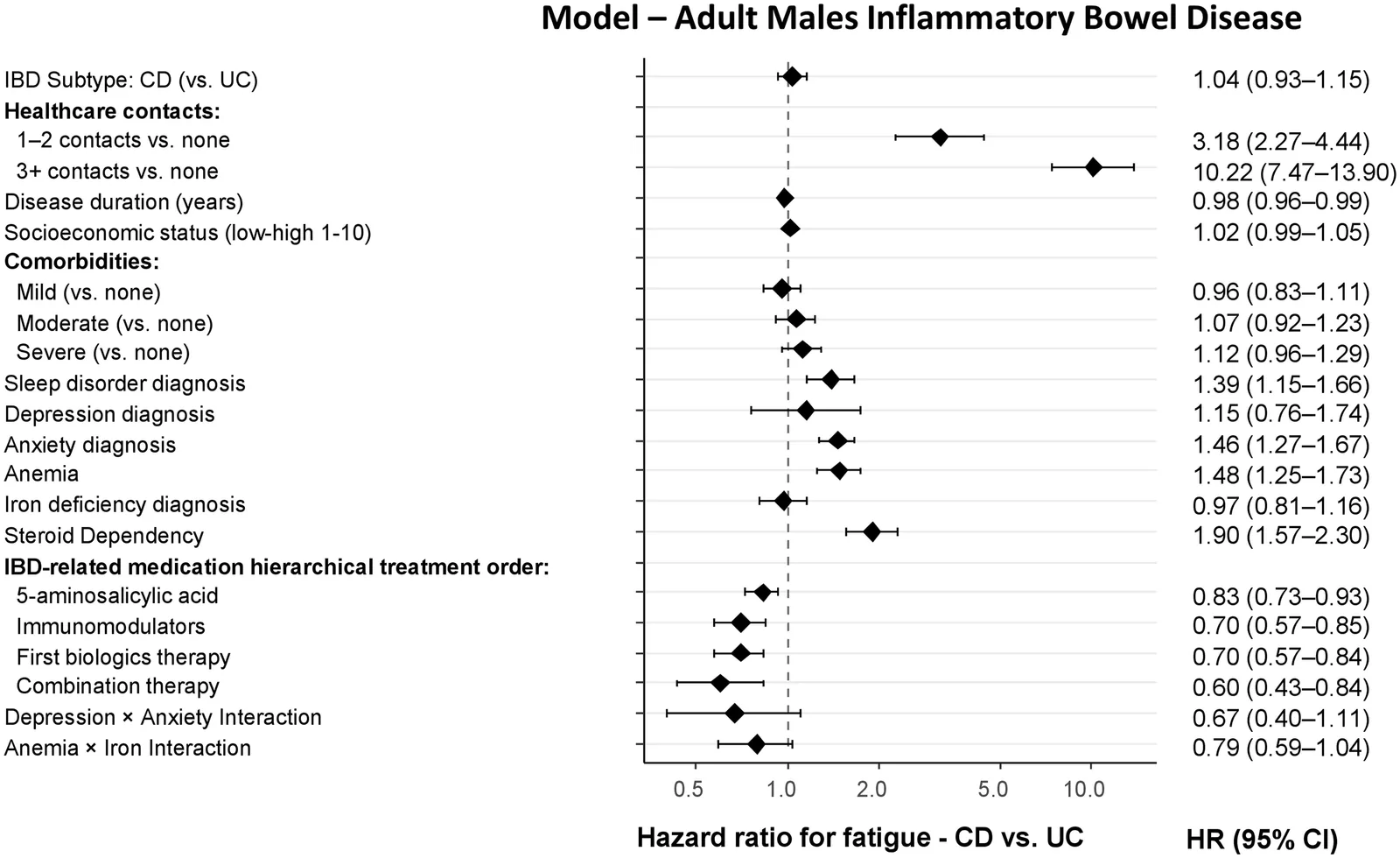
Adjusted risk factors associated with physician documented fatigue in adult males inflammatory bowel disease (IBD) patients. Covariates included: IBD subtype: Crohn’s disease versus ulcerative colitis, healthcare contacts: 1-2 versus none, healthcare contacts: ≥3 versus none, disease duration, years, socioeconomic status, scale 1-10, comorbidity burden:- mild/moderate/severe versus none, sleep disorder diagnosis, depression diagnosis, anxiety diagnosis, anemia diagnosis, iron deficiency diagnosis, steroid dependency, IBD-related medication, depression × anxiety interaction, anemia × iron deficiency interaction. HR, hazard ratio.

**Figure 3:**
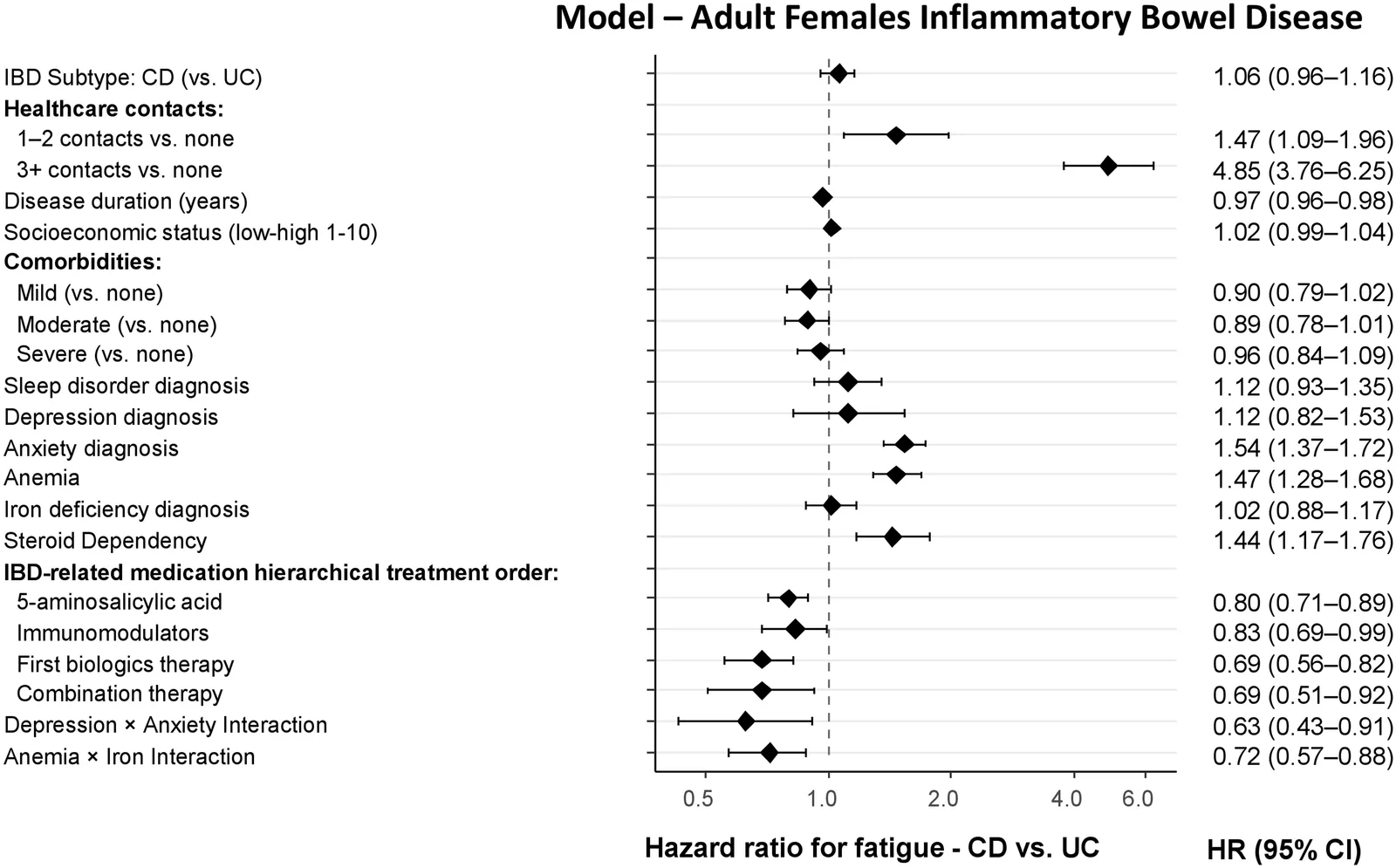
Adjusted risk factors associated with physician documented fatigue in adult females inflammatory bowel disease (IBD) patients. Covariates included: IBD subtype: Crohn’s disease versus ulcerative colitis, healthcare contacts: 1-2 versus none, healthcare contacts: ≥3 versus none, disease duration, years, socioeconomic status, scale 1-10, comorbidity burden:- mild/moderate/severe versus none, sleep disorder diagnosis, depression diagnosis, anxiety diagnosis, anemia diagnosis, iron deficiency diagnosis, steroid dependency, IBD-related medication, depression × anxiety interaction, anemia × iron deficiency interaction. HR, hazard ratio.

## Discussion

We found higher rates of documented fatigue among patients with IBD in all age groups compared to non-IBD matched controls (16.4% vs. 11%). These rates are lower than reported in a systematic review by D’Silva et al, in which the prevalence of self-reported fatigue in adults with IBD ranged across studies from 24% to 87%,^5^ including studies reporting prevalence of 72% during active disease, and 47% during remission. More recent studies report similar rates of 23%-70%.^30–33^ There is limited information on pediatric IBD, with only 3 studies reporting fatigue rates ranging from 22% to 78%.^6–8^ The lower fatigue rates reported here could be interpreted as under-reporting by physicians (as compared with patient-reported rates). A study exploring healthcare professionals’ perspectives, identified a gap in clinicians’ knowledge and appreciation of the complexity and full impact of IBD-related fatigue.^34^ Other studies have discussed physician under-recognition of fatigue among patients with IBD,^35^^,^^36^ and Lesage et al. reported that fatigue was considered a daily concern by 66% of patients, compared with only 36% of physicians.^37^ Alternatively, physician-reported fatigue may represent clinically significant or more severe cases that prompt specific medical attention. Notwithstanding the evident problems of inconsistent and under-reporting, our nationwide cohort was less prone to selection bias as it also included the mildest IBD cases not typically treated in tertiary centers from which the patients in the aforementioned studies were enrolled.

In the present cohort, physician-documented fatigue was more frequent among patients with IBD than among patients with other chronic conditions examined. However, comparative studies using patient-reported fatigue measures have yielded heterogeneous results, with substantial overlap between IBD and other chronic diseases.^38–40^ These findings could suggest that disease-specific factors in IBD, including nutritional,^14^^,^^41^ or microbial and metabolic aspects,^42^ may contribute to fatigue rather than attributing fatigue to the presence of a chronic disease. SES differed between patients with IBD and controls. This may influence healthcare utilization and symptom documentation; therefore, residual confounding related to this imbalance cannot be excluded.

Among the patients with IBD, we showed similar fatigue rates between CD and UC, while some previous publications reported higher rates of fatigue in CD^31^^,^^43^ Similar to other studies, we showed associations between fatigue and anemia,^44^ anxiety,^45^^,^^46^ depression^13^^,^^47^ and sleep disorders.^30^^,^^31^^,^^48^ In accordance with previous studies, fatigue was more common among females.^5^^,^^30^ Anemia, anxiety, and ≥3 healthcare contacts were independently associated with fatigue among males, females, boys, and girls. The particularly strong association with anxiety in the pediatric subgroup is consistent with recent pediatric data highlighting the importance of psychosocial and transdiagnostic factors in IBD-related fatigue.^45^ Although frequent healthcare contacts may partly reflect greater underlying disease burden, increased healthcare utilization could also precede and contribute to the recognition of fatigue, as patients experiencing persistent or poorly defined symptoms may seek repeated medical evaluation before fatigue is formally documented. While steroid-dependency was independently associated with fatigue among adults, potentially reflecting greater disease severity, other treatment-related findings did not show a consistent relationship between treatment intensity and fatigue, as biologic and combination therapy were associated with a lower hazard of fatigue despite their frequent use in patients with more severe disease. Anemia was independently associated with physician-documented fatigue in our cohort and may represent an important modifiable contributor. Notably, 11.8% of fatigue diagnoses occurred within ±3 months of an anemia diagnosis, indicating temporal proximity between the 2 in a subset of patients. In contrast, the association between iron deficiency in the absence of anemia and fatigue is less consistent, with Goldenberg et al. reporting no significant association.^49^ Greater laboratory-derived disease severity at diagnosis was not associated with a higher likelihood of subsequent fatigue, in contrast to some previous publications.^5^^,^^30^ However, as laboratory measures were not assessed longitudinally, we could not determine whether fatigue occurred during periods of active disease or deep remission. We report higher rates of sickness absence days among patients with fatigue and more health contact, implicating an economic burden associated with fatigue.^50^ Conversely, higher utilization of the health system could increase the likelihood of recording fatigue when it exists. Furthermore, administrative documentation patterns may be influenced by external incentives; for instance, patients seeking formal documentation for insurance claims or social security benefits may actively report fatigue, thereby increasing the probability of its official recording. Yet, this would not be limited only to the patients with IBD and would also appear in the non-IBD controls with other comorbidities. For the overall ever/never comparison, fatigue codes recorded within the 2 years preceding the index date were retained to capture possible early manifestations of IBD during the diagnostic-delay period. Although the same index date and exclusion criteria were applied to matched controls, healthcare-contact intensity may have differed during this pre-diagnostic period, potentially affecting opportunities for fatigue documentation; however, the higher frequency of physician-documented fatigue in IBD persisted across comorbidity strata. Another possible limitation is that fatigue ICD9 codes were not validated in the epi-IIRN cohort in the same way that IBD codes have been validated, nor against patient-reported fatigue. Thus, our outcome represents documented fatigue in the electronic health record and should not be interpreted as a validated measure of the prevalence of fatigue experienced by patients. A prospective validation study linking routinely collected electronic health-record data with validated patient-reported fatigue measures, using appropriate consent, would therefore be valuable to determine how well physician-documented fatigue reflects the patient-experienced burden. In addition, we included only fatigue codes recorded within diagnostic code fields and not fatigue documented in free-text clinical notes. It is therefore possible that some physicians documented fatigue in clinical notes without assigning a corresponding diagnostic code, which may have contributed to the lower observed fatigue rates. On the other hand, major strengths of the epi-IIRN include its large sample size and nationwide, population-based design, which reduces the potential for referral-related selection bias.

In summary, our study demonstrates that real-world, unselected physician-documented fatigue is more prevalent in patients with IBD compared with matched non-IBD controls. This difference persisted across strata of comorbidity burden. However, the fatigue rate in medical records seems lower than that reported from studies based on self-reporting, possibly indicating that this captures the “tip of the iceberg” of fatigue burden among patients with IBD. Within the IBD cohort, we identified several potential predictors of fatigue including clinical factors, medication use, and psychological characteristics.

## Supplementary Material

otag116_Supplementary_Data

## Data Availability

Access to the anonymized data utilized in this study, including protocol and analytic code will be granted upon a reasonable request to the corresponding author.
