# Supplementary material for: Physician documented fatigue among adults and children with inflammatory bowel disease: a nationwide routinely collected health data study from the epi-IIRN
Source: Crohns Colitis 360. 2026 Oct 12;8(4):otag116. doi: 10.1093/crocol/otag116 (PMC13722277; doi:10.1093/crocol/otag116)
Supplement: Supplementary file 1 [file otag116_supplementary_data.zip › Fatigue_epi_IIRN_manuscript_supp_tables_30.9.26_clean.docx]

**Supplementary Tables**

Supplementary Table 1: List of physician-documented fatigue related codes

MALAISE AND FATIGUE

OTHER MALAISE AND FATIGUE

CHRONIC FATIGUE SYNDROME

780.7 - Malaise And Fatigue Asthenia NOS; Lethargy; Postviral (asthenic) syndrome; Tiredness

780.79 - OTHER MALAISE AND FATIGUE

780.71 - CHRONIC FATIGUE SYNDROME

MALAISE AND FATIGUE (ASTHENIA/LETHARGY/TIREDNESS)|EXHAUSTION|FATIGUE|LETHARGY|MALAISE|MALAISE & FATIGUE|MALAISE AND FATIGUE|POSTINFLUENZAL ASTHENIA|WORN OUT

MALAISE AND FATIGUE-WEAKNESS

NEURASTHENIA (FATIGUE)

Supplementary Table 2: IBD-related medications categorized by hierarchical treatment order at each disease quarter

| **Order** | **Description** |
| --- | --- |
| 1 | No treatment |
| 2 | 5-aminosalicylic acid |
| 3 | Immunomodulators |
| 4 | First biologics therapy |
| 5 | Combination therapy – biologics therapy together with immunomodulators defined as starting from 120 days of simultaneous use / biologic switch |

Supplementary Table 3: List of Anemia and iron deficiency-related codes

285.9 - Anemia, unspecified

285.29 - Anemia of chronic illness

281.9 - Vitamin B deficiency anemia, unspecified

281.2 - Folate deficiency anemia

281.1 - Pernicious anemia

280.9 - Iron deficiency anemia, unspecified

Supplementary Table 4: Physician-documented fatigue and other related comorbidities in the pediatric IBD population (numbers (%), median (IQR) or means ± standard deviations are presented as appropriate)

| **Variable** | **Pediatric patients with IBD^a^ with no physician documented fatigue (n=4,760)** | **Pediatric patient with IBD^a^ with a physician documented fatigue (ever) (n=469)** | **SMD^b^** | **OR^c^ (95%CI^d^)** |
| --- | --- | --- | --- | --- |
| **IBD Subtype, CD^e^** | 3,117 (65.5%) | 334 (71.2%) | 0.123 | -- |
| **Sex (Male)** | 2693 (56.6%) | 237 (50.5%) | 0.121 | -- |
| **Age at Diagnosis** | 13.33 ±3.75 | 13.70 ±3.33 | 0.104 | -- |
| **Sickness absence days^f^** | 52.56 ±65.30 | 64.40 ±68.22 | 0.177 | -- |
| **Socioeconomic status** | 5.94 ±2.09 | 6.05 ±2.13 | 0.048 | -- |
| **Anemia** (ever/never) | 1,565 (32.9%) | 214 (45.6%) | -- | 1.71 (1.41-2.07) |
| **Anxiety** (ever/never) | 410 (8.6%) | 92 (19.6%) | -- | 2.59 (2.02-3.32) |
| **Depression** (ever/never) | 87 (1.8%) | 16 (3.4%) | -- | 1.90 (1.10-3.26) |
| **Iron Deficiency** (ever/never) | 2,209 (46.4%) | 215 (45.8%) | -- | 0.98 (0.81-1.18) |
| **Sleep Disorder** (ever/never) | 140 (2.9%) | 21 (4.5%) | -- | 1.55 (0.97-2.47) |
| **Disease severity at diagnosis^g^** |  |  | 0.094 | -- |
| Mild | 768 (16.1%) | 84 (17.9%) | -- | 0.88 (0.69-1.12) |
| Moderate | 2,929 (61.5%) | 267 (56.9%) | -- | 0.85 (0.67-1.08) |
| Severe | 1,063 (22.3%) | 118 (25.2%) | -- | 1.21 (0.98-1.49) |

^a^ IBD – Inflammatory Bowel Disease

^b^ SMD (standardized mean difference) reflects the effect size and not only statistical significance which is more appropriate in large datasets. SMD is considered significant when> 0.1^25, 26^

^c^ OR – Odds Ratio

^d^ CI – Confidence Interval

^e^ CD – Crohn’s disease

^f^ Starting 2 years prior to IBD diagnosis till the end of follow-up

^g^ By clustering analysis of blood tests available at diagnosis^24^

Supplementary Table 5: Predictive covariates of fatigue among pediatric patients with IBD (boys and girls) - multivariable Cox models

| **Covariates** | **Boys (HR, 95%CI)** | **Girls (HR, 95%CI)** |
| --- | --- | --- |
| IBD subtype (CD) | 0.79 (0.52-1.19) | 0.91 (0.61-1.36) |
| Disease duration (years) | 0.95 (0.90-0.99) | 0.94 (0.89-0.99) |
| Socioeconomic status (low to high 1-10) | 1.09 (0.99-1.19) | 1.01 (0.91-1.10) |
| Comorbidity (CCC*) | 1.44 (0.95-2.17) | 1.22 (0.78-1.89) |
| Healthcare contact 1-2 (vs. none) | 0.91 (0.25-3.25) | 3.32 (0.72-15.2) |
| Healthcare contact ≥3 (vs. none) | 4.2 (1.49-11.70) | 7.21 (1.74-29.7) |
| Anemia diagnosis | 1.86 (1.06-3.25) | 3.21 (1.92-5.33) |
| Iron deficiency diagnosis | 1.07 (0.64-1.77) | 0.95 (0.54-1.59) |
| Anxiety diagnosis | 3.06 (1.62-5.77) | 4.14 (2.38-7.17) |
| Depression diagnosis | 2.75 (0.37-20.10) | 1.21 (0.27-5.39) |
| Sleep disorder diagnosis | 1.03 (0.32-3.30) | 1.05 (0.25-4.30) |
| Steroid dependency | 0.75 (0.35-1.58) | 1.07 (0.54-2.13) |
| IBD related medication (vs all hierarchies below) |  |  |
| 5-aminosalicylic acid | 0.52 (0.29-0.92) | 0.54 (0.31-0.95) |
| Immunomodulators | 0.86 (0.52-1.42) | 0.63 (0.35-1.10) |
| First biologic therapy | 0.63 (0.35-1.11) | 0.92 (0.51-1.61) |
| Combined therapy | 0.89 (0.42-1.84) | 0.78 (0.34-1.72) |
| Anxiety X Depression | 0.24 (0.01-4.17) | --** |
| Anemia X Iron deficiency | 0.97 (0.44-2.14) | 0.53 (0.24-1.17) |

* CCC – the Pediatric Chronic Complex Conditions
** The anxiety × depression interaction could not be estimated in girls because of sparse events; both variables were therefore entered as separate main effects.

SUPPLEMENTARY FIGURES LEGENDS

Supplementary Figure 1: Rates of Physician Documented Fatigue codes given by primary physicians and specialists in both patients with inflammatory bowel disease (IBD) and non- IBD controls

Supplementary Figure 2: Time to first Physician Documented Fatigue code in Crohn’s disease and ulcerative colitis patients from diagnosis (A. adults and B. pediatric)

Supplementary Figure 3: Time to first Physician Documented Fatigue code in males and females with inflammatory bowel disease (IBD) (A. adults and B. pediatric)
